# Collaborative, individualised lifestyle interventions are acceptable to people with first episode psychosis; a qualitative study

**DOI:** 10.1186/s12888-018-1692-0

**Published:** 2018-04-25

**Authors:** Rebecca Pedley, Karina Lovell, Penny Bee, Tim Bradshaw, Judith Gellatly, Kate Ward, Adrine Woodham, Alison Wearden

**Affiliations:** 1 Division of Nursing, Midwifery & Social Work, The University of Manchester, Manchester Academic Health Science Centre, Jean McFarlane Building, Oxford Road, Manchester, M13 9PL UK; 2 Mersey Care NHS Foundation Trust, Ashworth High Secure Hospital, Ashworth Research Centre, Parkbourn, Maghull, Liverpool, L31 1HW UK; 3 Division of Population Health, The University of Manchester, Williamson Building, Oxford Road, Manchester, M13 9PL UK; 4 Division of Psychology and Mental Health, The University of Manchester, Manchester Academic Health Science Centre, Coupland 1 Building, Oxford Road, Manchester, M13 9PL UK

**Keywords:** Acceptability, Antipsychotic medication, Early intervention, Healthy living, Psychosis, Weight-gain

## Abstract

**Background:**

The adverse impact of unhealthy lifestyle choices and the prescription of antipsychotic medications contribute to weight gain, poor cardiovascular health and reduced life expectancy for people with psychosis. The present study aimed to explore the acceptability and perceived outcomes of a lifestyle intervention designed to prevent or reduce weight gain in people with first-episode psychosis.

**Methods:**

This was a qualitative study using a data-driven approach. People recovering from first-episode psychosis recruited from UK early intervention services and taking part in the active arm of a randomised controlled trial of a lifestyle intervention (the InterACT trial), were interviewed using a semi-structured interview schedule. Interviews were transcribed verbatim and analysed using Framework Analysis.

**Results:**

Participants valued the collaborative and individualised approach taken by the intervention deliverers, and formed high quality relationships with them. Aspects of the intervention that were positively appraised included goal setting, social opportunities, and progress monitoring. Benefits of the intervention, including increased levels of exercise; improved diet and physical health; increased psychological wellbeing (e.g. confidence, self-esteem); and improved social relationships, were identified by participants, independent of actual weight loss.

**Conclusions:**

Future interventions should ensure that workers have the skills to form high quality relationships with users, and to individualise the intervention according to users’ needs and preferences. Future trials that test healthy living interventions should consider supplementing physical outcome measures with wider psychosocial outcome assessments, in particular social relationship quality, psychological wellbeing, self-esteem and self-efficacy.

**Trial registration:**

Current Controlled Trials: ISRCTN22581937. Date of registration: 27 October 2010 (retrospectively registered).

## Background

People with serious mental illness such as psychosis are at increased risk of weight gain and cardio-metabolic changes which adversely impact their physical health and life expectancy [1–4]. While some of the risk is due to lifestyle factors [5], antipsychotic medications also play a role [4]. People with first-episode psychosis are at particular risk of the adverse effects of antipsychotics, with evidence of unhealthy metabolic changes developing within just 6–8 weeks of commencing treatment [2]. One large open randomised controlled trial found that the proportion of individuals with first-episode schizophrenia, schizophreniform or schizoaffective disorder gaining > 7% of their bodyweight one year after commencing antipsychotics ranged from 37-86%, depending on the antipsychotic prescribed [6]. The long-term consequences of these negative changes have been found to contribute to physical illnesses such as circulatory disease [7] and coronary heart disease; ultimately leading to a reduced life expectancy of 20% compared to the general population [8].

In recent years, a variety of ‘lifestyle interventions’ have been developed with the aim of improving the physical health of people with psychosis and other serious mental illnesses [9]. Lifestyle interventions aim to adjust dietary and / or physical activity behaviours, usually based on psychological theory to achieve behaviour change. A recent meta-analysis examining the effects of lifestyle interventions on weight management for people with psychosis found that effect sizes were moderate for interventions aimed at weight loss and high for interventions targeting weight gain prevention [10]. These benefits were sustained up to 6-months post intervention. The authors however noted that the quality of studies was poor with only 4 of the 25 RCTs included judged to be high quality [10].

Qualitative evaluation of the experience of lifestyle interventions for people with first-episode psychosis has been limited. Firth et al. [11], reporting on findings from a qualitative study on the views of individuals undergoing a 10-week exercise intervention, found that the intervention was perceived to be highly acceptable and beneficial to the participants’ psychological health, including their psychiatric symptoms. To the best of our knowledge, no other study has used qualitative methods to evaluate the acceptability of a lifestyle intervention for those with first-episode psychosis who are already overweight and at risk of physical health problems.

Using the United Kingdom Medical Research Council (MRC) framework for complex interventions [12, 13], we developed a new healthy living intervention to reduce weight gain in people with recent-onset psychosis [14]. This intervention was tested in an exploratory randomised controlled trial (RCT) comparing the lifestyle intervention with treatment-as-usual provided by two UK based Early Intervention Services [15, 16]. Although the intervention was not effective in modifying its primary outcome (weight gain reduction) [15], engagement was high (77.7% completed at least six of the eight sessions).

Evaluation of the acceptability of a new intervention is necessary prior to a phase III definitive RCT, to enable the identification of any necessary modifications to optimise treatment [13, 17]. Without demonstrable acceptability, it is unlikely that new treatments will be integrated and normalised in practice. This qualitative study, nested within an RCT, examined the views of those who received the intervention with an aim to explore its acceptability and perceived impact.

### Intervention

The InterACT (INTERvention to encourage ACTivity, improve diet, and control weight gain) healthy living intervention was developed from a synthesis of findings from a series of interconnected studies, incorporating a systematic review and qualitative exploration of the perspectives of service users, carers and health professionals (for further details, see [14–16]). The intervention was underpinned by the Common Sense Model [18], a theoretical framework which posits that individuals’ personal beliefs about a health problem (e.g. its controllability, causes and consequences) are important in determining how they respond to a given condition, i.e. in the case of weight gain, whether the individual takes steps to reduce the weight gain through diet and exercise.

The intervention incorporated eight one-to-one sessions with one of four specifically trained Support Time Recovery (STR) workers, delivered across increasingly spaced intervals over a one-year period. STR workers are unqualified staff members who provide support and advice to service users, for example by providing smoking cessation guidance and providing help in accessing healthcare and other community resources. The STR workers delivering the intervention underwent a three-day intensive training course (supported by a training manual- available on request from the first author), delivered by members of the research team and co-facilitated by several service-user partners. The course provided information about psychosis and the effects of antipsychotic medication, diet and physical activity, and the theoretical framework for the intervention. Trainees used vignettes and role plays to practice aspects of the intervention, including understanding beliefs about weight gain, providing information and correcting unhelpful beliefs, eliciting goals, goal-setting and feedback.

Intervention sessions involved the elicitation of health beliefs and development of personalised goals and action plans, supplemented by healthy living education delivered by an STR worker and through accompanying materials, including an ‘InterACT’ healthy living booklet and website [14–16]. Socially inclusive group sports activities and family involvement in intervention sessions were offered as optional additional components of the intervention. STR workers received supervision every 2 weeks, delivered by a research team member (TB) with professional training in adult (physical) and mental health nursing.

## Methods

Ethical approval was obtained from Cumbria & Lancashire B Research Ethics Committee (09/H1016/20). The design was a qualitative study using a data-driven approach.

### Participants

All individuals in the exploratory RCT had experienced a recent episode of psychosis (within 3 years preceding the trial) and had been recruited with a minimum BMI of 25 or more (24 if of South Asian origin). Fifty four individuals were randomised to the intervention of which 48 completed the 12-month follow-up and were invited to complete a qualitative interview to explore their experiences of the intervention. Of these, 25 consented and participated.

### Procedure

Trial participants were invited to participate by a research assistant (in person or by letter) following the completion of their final 12-month trial assessment. Individuals providing written informed consent were interviewed using a semi-structured interview schedule, which was developed through team discussion (see [16] for full version) and explored views on the materials, context, delivery mode and outcomes of the intervention. Interviews were conducted by an independent qualitatively-trained, master’s degree level researcher (KW), at the participant’s home or other location of their choice. Audio-recorded interviews were transcribed verbatim. Where recording was refused (*n* = 2), detailed notes were taken.

### Analysis

Analysis was undertaken using the principles of Framework Analysis [19]. The first author developed close familiarity to the data by listening to the audio-recorded interviews (whilst also checking the accuracy of transcription) and through repeatedly reading the transcripts. Recurrent themes relating to the acceptability of the intervention were identified by RP, with further validation through group discussion with other team members, who independently identified key themes within a sub-set of the transcripts. The resulting themes and ideas were organised into a conceptual framework, which were grouped by higher-order themes and subthemes and assigned a number. The Framework was applied by hand (sometimes referred to as ‘indexing’) to each of the transcripts on a line-by-line basis. A set of thematic charts were generated using Microsoft Excel (one per theme); with each row representing an individual participant and each column representing themes and sub-themes. By summarising indexed data from the transcripts into the relevant thematic chart, it was possible to view the whole data set across all participants. The completed charts were further analysed and through mutual discussion within the team (AW,KL,TB,JG,PB,Awo), themes and sub-themes were refined. The process of analysis involved moving from four higher order themes, each with respective sub-themes (range of 5 to 10 per theme) to a more analytically refined final set of three higher order themes, with 11 sub-themes in total. The final set of themes and sub-themes will be described in the results section.

## Results

The sample comprised 15 males and 10 females with a mean age of 26.5 (SD 5.7) and an age range of 17 to 39. Interviews lasted a mean of 41.4 min (SD 10.5). Participants attended a mean of 7.3 intervention sessions (SD 1.2; range 4–8); compared with a mean of 5.8 (SD 2.6) sessions completed by the 23 eligible participants who did not participate. BMI change in the interviewed sample was comparable to the trial intervention arm as a whole.

Three main themes were identified: Creating momentum for change (factors facilitating lifestyle changes, The centrality of the STR worker role (their role in personalisation of the intervention and meaningful relationship development), and Perceived intervention impact; beyond physical benefits (the range of benefits perceived by participants). Quotes are presented to illustrate each theme together with details of patient ID (Pt.x).

### Creating momentum for change

Participants spoke about the factors facilitating positive lifestyle changes that had overcome barriers of low motivation, confidence and apathy towards physical health.

### Increasing motivation through goal-setting

Participants described how setting goals motivated positive changes to lifestyle and provided a framework by which to gradually ‘step up’ adjustments in a manageable way:

*“ … Once you reach the three-month goal, it gives you this spur to go for the six months…”* (Pt.8)

Although most were positive about goals, some were more ambivalent, stating that they could not recall their goals. One indicated they did not want to achieve the goals set, suggesting ineffective participant involvement in the goal-setting process.

### The motivational role of self-monitoring

Participants spoke of opportunities for monitoring and assessment, such as food and exercise diaries, weight measurements and rating goals.

The view of one individual who was initially sceptical about the benefits of keeping an exercise diary changed over time:

*“…at first I thought, ‘What a lot of old bollocks’ to be truthful. But then…within a couple of weeks I could see, from obviously page one to, like three or four weeks down the line, you know, it dropped, er, you know, I was doing more and more*…” (Pt.9)

For some, assessment and monitoring helped to develop a new understanding of their own behaviour, e.g. by using a food diary, one participant identified that their weight gain may be due not only to medication, but also due to dietary intake. Whilst weight measurements were not a component of the intervention, the opportunity to be weighed was particularly valued by those who did not own scales or perceived the trial scales to be highly accurate. The following participant explained that the shock of learning of their current weight gain at the point of entering the trial had created a strong motivation to improve their lifestyle behaviours:

*“…Ever since that first weigh in, it made me more conscious of the fact that, oh, I know I’m, you know, I must go out and get some exercise. Even when I’m having a bad day…”* (Pt.15)

### Developing a healthy living knowledge base

The intervention brought about an increased understanding of the importance of good physical health. This was particularly pertinent where individuals had focused their attention on their mental health:

*“…it reminded you about the food stuff you need to be careful what you eat, cos if you’re in a mental mess about the psychosis, sometimes you just forget about the food…”* (Pt.3)

Participants’ interest or enthusiasm for living a healthier lifestyle increased as a result of being involved in the intervention. One individual described how the information on healthy living had acted as a catalyst, leading them to experiment:

*“…in the super foods aisle…we just went nuts and just bought everything that we could, just to try it out in different things…I think it was part of curiosity about foods that we had never had…”* (Pt.25)

Through attempting new behaviours, participants realised that the activity was more enjoyable than previously assumed, increasing the likelihood that it would be continued.

The intervention booklet was particularly valued by those who had received no prior opportunity to learn about healthy living. The following participant explained how their knowledge of healthy living had been very limited until taking part in the intervention:

*“..I didn’t know anything about it [calories and the impact of weight gain], but now, I’m very, very aware of what is going on* and it’s helped me, because of that programme*”* (Pt.19)

Although those with good existing knowledge found the information less useful, many regarded the information as reinforcing or expanding their knowledge:

*“…I knew about [nutrition] already to a certain degree but I wasn’t kind of practicing, so kind of more backup in terms of the more you drum something in, the more it’s kind of going to sink in and you’re more likely to repeat that in your own actions.”* (Pt.12)

Some were unwilling or unable to engage with the booklet, for example due to literacy problems. These individuals felt that information delivered in an alternative format, (e.g. video), would be preferable. For some, face-to-face work with their STR worker remained most effective:

*“…what really worked for me was actually seeing (STR)…and talking to somebody, do you know what I mean, and getting those tips; rather than somebody coming and just give me a handout, handout…“you work on that, I’ll see you on a month’s time”* (Pt.9)

### Facilitating social support

Although few participants involved their family or friends in sessions, they reported that they provided support in a variety of ways, for example by enquiring about their progress, accompanying them on walks or improving their diet:

*“…they [friends] sort of changed with me, to sort of show well, we can do it as well, so you can.”* (Pt.14)

There was some evidence that in trying to help the participant, family and friends also developed more interest in healthy living themselves; a number described passing on the information gained to their family members. One participant described how their newly developed interest in cooking had rekindled a closer relationship with their mother:

*“…my mum’s been very, like, ringing me up going, are you still eating healthy, and stuff, and that’s actually got me close to my mum again because me and my mum have been very distant…”* (Pt.2)

Another described how joining a walking group arranged by their STR worker had motivated them:

*“…I was not walking far, because I was getting bored and, then, I was feeling tired, because, I was overweight and tired, feeling tired. So, to walk, as a team, it helped me so much.”* (Pt.19)

Other individuals recognised the benefits of group activities, though only wanted to participate if groups were open to all, rather than being mental health specific. A significant barrier to involvement in mental health specific groups appeared to be the concern that this would compromise confidentiality. Some mistakenly assumed that they would be forced to share details about their mental health:

*“…I don’t like group work, do you know, I’m not into, like, sitting round in a circle and going, my name’s ‘Dave’[pseudonym]…”*(Pt.9)

### Developing confidence

For individuals who suffered loss of self-esteem and confidence, the availability of a supportive individual was vital in giving them the confidence to attempt lifestyle changes.

*“…I’ve specifically lacked confidence since the breakdown. And he gave me a lot of confidence which I, which I needed. I needed someone just to perk me up a bit. Just to say, you know, good job, you know. And that meant a lot to me.”* (Pt.20)

Low confidence created a significant barrier to accessing sporting activities in the community, which could be overcome by the support of the worker:

*“…if you’re gone all your life from being a size eight and all of a sudden you’re like a size 14 and nearly going into a 16 the last thing you want to do is get into a swimming costume. But I just decided…well I had an interview with [STR] and I decided that was what I want to work up to I think it was days later I just rang [STR] and said I’ve bought a swimming costume let’s just get it over with...”*(Pt.16)

By initiating activities with STR support, some individuals built sufficient confidence to continue the activity independently. However, some reported not receiving the opportunity to attend joint sporting activities with their worker. One such individual explained how their confidence could have been boosted by this opportunity:

“…*I think that would have just given me the first step in kind of doing the goals because if [STR] said like ‘oh go to the gym’ so and so like twice a week or something I wouldn’t have gone there myself, because at the time I had like no confidence and I needed someone there with me to help me like communicate with people, because I found it really difficult*.” (Pt.21)

### The centrality of the STR worker role

The role of the STR worker in personalising the intervention and their ability to form meaningful relationships with the participants was central to intervention acceptability.

### Individualised approach

STR workers were trained to explore the individual’s existing health beliefs and to develop goals and action plans accordingly. They were appraised positively for the way in which they sought further information and resources for the participant as required, involved individuals in goal setting and respected participant’s wishes to decline or pursue activities. Workers who were able to assess the individual’s current lifestyle and give personalised tips and advice, were regarded as helpful and motivating:

*“…my diet was still as if I was working full time…[STR] made me realise, because I’m not working full time, I’m not working such a physical job…that…you’ve got to change some of your habits.”* (Pt.15)

Two participants perceived their STR workers as being less responsive and had poorer relationships with them. One individual felt that their worker had not spent enough time getting to know them, resulting in a feeling of their wishes being ignored:

*“…that was quite a bit rude, like not finding out for me or not…that’s like not caring. Just wanting me to do her thing, and that’s all that mattered.”* (Pt.4)

This individual felt that the failure to understand their difficulties had led to the worker being too pushy. In contrast, most participants described a worker who provided an appropriate degree of motivation:

*“…he never pushed me or anything like that, it was at my own choice, at my own speed, but he just helped obviously where he thought it was necessary.”* (Pt.7)

### Relationship quality

Most participants described high quality relationships with their STR worker, describing them as friendly, easy to talk to and professional. It was clear that many participants enjoyed and looked forward to their meetings. Some participants preferred a worker of a similar age, matched gender, or who shared their objective to lose weight. Sharing common ground encouraged openness in the relationship:

“*I think he was good as well because he was like just a couple of years older than me, not like forty odd and I’m like, oh God, I can’t tell him I went home pissed the other weekend*…”(Pt.22)

Indeed, one participant who had a poor relationship with their worker suggested that their relationship could have been improved if they had been the same gender.

Many participants appreciated the flexible and understanding nature of their worker, particularly when mental health problems were challenging. They talked about the non-judgemental and empathic attitude of their worker, which one participant described positively having confessed to eating ‘fast food’:

“…*she said well next time you are in the kebab shop get a chicken kebab trust me they are delicious but there is so much less rubbish in it….I was like yeah and that stuck…stuff like that, it was never kind of whoa, it was very supportive*” (Pt.25)

### Perceived intervention impact; beyond physical benefits

Although the achievement of weight loss was an important outcome for participants, acceptability also derived from the behavioral and wider psycho-social benefits precipitated by intervention involvement.

### Physical impact

Although only 56% of those interviewed had lost weight, participants were often positive about their weight outcomes. As well as those who reported significant reductions in their weight, a number expressed their relief in having prevented any further weight gain:

“*It’s [weight] been stable for maybe like six months which is like a massive achievement because the amount I put on was like ridiculous…I don’t know I just I am like really grateful to the study because I think otherwise I don’t know what I would have looked like then*…” (Pt.21)

Some explained that they had failed to lose weight despite having made behavioural changes:

*“I’ve reached most of it [goals], I haven’t reached the weight one yet but that’s…that will come along at some other point but, like, I’ve done my eight miles of walking a day*…” (Pt.2)

Participants additionally described a range of physical health benefits associated with the intervention, e.g., increased fitness, reduced headaches, improved skin and nails.

### Behavioural impact

While they may not have experienced a decrease in BMI, many participants nevertheless perceived having improved their lifestyle behaviours. Many described increasing physical activity, either through engaging sports activities or by being more active in their daily lives. One participant, who initiated walking and bi-weekly gym attendance, explained how these activities had become integrated into daily life:

*“I used to get tired and tell him…dad I can’t, you know, I can’t carry on. But now it’s like it’s become like more of my life doing walking, eating well”* (Pt.6)

Participants also described a range of changes to their dietary behaviours, particularly in making healthier choices of food, doing more ‘home cooking’, controlling the quantity of food consumed and changes to eating patterns. It was notable that rather than adhering to a diet aimed solely at reducing weight (e.g. cutting out ‘treats’), participants’ increased understanding of nutrition resulted in efforts to maintain a well-balanced diet:

“…*it’s much easier to go somewhere like [supermarket] and get nuggets in a bag and chuck them in the oven. Whereas now I’m actually able to cook things from scratch. I’ve learned a lot more about…different food groups, introducing things like, rice, pasta, different meats, vegetables …”* (Pt.8)

Although almost all participants described making lifestyle changes, a small number of individuals achieved considerably less, citing barriers to engagement such as their mental health problems.

Many reported that they were continuing to adopt their lifestyle changes at the end of the intervention and appeared to be motivated to maintain changes or even to set themselves more ambitious challenges:

“…*all my goals I’ve gone past now but now I’ve just changed the way I am now. Like I’m always thinking, oh what can I start doing now or, oh kayaking should I do that? Yeah so I’ve already changed. I’m setting myself goals but not realising them…*” (Pt.22)

### Psychological benefits

Participants described a number of psychological benefits associated with an improved sense of wellbeing, e.g. improved mood, better ‘*frame of mind*’, more vitality/increased energy etc. Some perceived that their improved wellbeing had been produced as a direct consequence of particular behavioural changes e.g. doing more exercise or eating more healthily:

“*I think it has improved my mood a bit because, um, one of the things when I was eating really unhealthily is I’d feel happy when I ate the food but then at the end of the day I’d feel really rubbish and I did get more things like headaches*…”(Pt.3)

However, other psychological benefits derived from a more complex mechanism whereby the outcome of behaviour change (particularly weight loss), had increased psychological wellbeing. Participants described feeling happier with their appearance, leading to benefits in terms of confidence and self-esteem:

“…*I lost weight, so, I can have a nice dress…my confidence is back, because when I talk to people, I don’t think that they’re going to look at how I look …”* (Pt.19)

Five participants reported having gained an understanding of how to control weight. This was particularly important to individuals who had previously felt that their weight gain had been uncontrollable. For one, the perception of increased control appeared to generalise to a feeling of greater self-efficacy:

“*It made me realise that I can do things and that I can be in control and I can…live my life the way that I want to live it, not run by my medication or my community nurse*…” (Pt.7)

Another, who had stopped taking their medication as prescribed, started taking it more regularly once they had gained control over their weight, leading to improvements in their mental health:

“…*I just didn’t want to take it [medication] because…I can’t deal with it. But then it’s like I started maintaining my weight, I think I feel a lot more motivated to take my medication so I do feel better because of that.” *(Pt.21)

### Social impact

Participants reported improvements to social relationships brought about by increased opportunities to socialise and improvements to the quality of relationships. The frequency with which participants saw friends/family had increased and participants also noted increased social opportunities when undertaking exercise. One participant explained how he had enjoyed interacting with other members of the community since taking up walking:

“ *On my walks, and stuff, you always bump into people, you’re always bumping into old people and couples and kids, especially riding bikes*…” (Pt.2).

Sometimes participants attributed their improved social relationships to increased confidence, energy or ‘*feeling better*’ as a result of the intervention. One felt that the opportunity to undertake joint sports activities with their STR worker had improved their mood and provided them with a new topic of conversation. Another felt that their relationship with the STR worker had played a significant role in improving their confidence to talk to others:

“…*I’ve got friends, which I didn’t before I went to college and …it made me find it easier to talk to them, because I was talking to [STR worker] and, then, I was talking to people who were in this thing [intervention], so it just helped me to talk more…”* (Pt.17)

## Discussion

A range of factors contributed to the acceptability of the InterACT healthy living intervention, central to which was the role of the STR worker, and in particular, his/her attention to the individual’s needs and preferences, e.g. giving personalised advice. The importance of individualising interventions has been echoed elsewhere [11]. It was notable that those aspects of the intervention that were less personally adaptable (e.g. healthy living booklet) were less positively appraised and modification of this intervention for further testing should seek to increase individualisation further through catering for other learning styles (e.g. online videos).

The person delivering the intervention was important; participants not only wanted a worker who was professional and ‘*nice*’, but someone whom they could relate to, who was tolerant and non-judgemental. This suggests that participants find it more acceptable to have a worker who they can form a meaningful relationship with. STR workers, individuals who are selected for their personal characteristics and previous experience of mental health services (either professional or personal) rather than any specific professional training, might be particularly suited to this role. Given the evidence in the literature associating a lack of positive staff-patient relationships in psychosis with poorer treatment outcomes [20], this point may be key.

The healthy living information not only provided knowledge of *how* to make changes but also explained the rationale for action, and engendered an understanding of the importance of physical health and lifestyle behaviours. In a narrative synthesis of incentives and barriers to lifestyle interventions for people with psychosis, knowledge gain was found to be a facilitator to engagement [21].

Participants also gained motivation from the format of the intervention, particularly in the opportunity to set goals and monitor current and on-going behaviours and outcomes. Future interventions may consider making weight monitoring a component of interventions as this was valued by many participants.

The importance of social support in promoting engagement has been reflected elsewhere [11]. Participants who found group activities acceptable, placed high importance on the opportunity this provided for social interaction. However, as our intervention development work suggested, not all individuals found group activities acceptable [14], and some held misconceptions about their content, e.g. expectations to share details of their mental health. It is possible that this view may have come from individuals’ experience of psychological therapy groups. It is important that group sports activities offered by mental health services recognise and overcome this barrier to participation. Our finding that participants garnered social support from friends and family outside the intervention sessions, recognised elsewhere in the literature [22], suggests that their direct involvement in sessions is not required, or necessarily desirable. Low confidence as a result of recent mental health problems and poor body image associated with weight gain were barriers which were sometimes overcome with positive encouragement from the STR worker in the context of accompanied sports activities. Some participants wanted more opportunity for joint sports activities; a feature that future interventions may benefit from offering. Participants perceived a range of positive effects including improved lifestyle behaviours, improvements to physical health, psychological wellbeing and relationships with others.

It is important to note that despite the perceived benefits reported by participants here, the trial found no significant difference in BMI between the intervention and control groups [15, 16]. It is possible that due to demand characteristics (e.g. to please the interviewer), our sample felt pressured to speak positively about the intervention. The employment of an interviewer independent to the trial/service team who encouraged positive and negative views reduces this possibility. It is also possible that those who found the treatment more acceptable were more likely to consent to interview; we know that they attended more sessions than those who did not consent to interview. Despite this, the impact of the intervention on BMI (primary outcome), was similar for both groups, suggesting that if acceptability was indeed elevated in the interview sample, this may relate to wider factors than BMI reduction, such as the perceived psychosocial benefits of the intervention reported in the findings. Finally, a limitation of the study is that we did not invite participants to take part in an interview until the end of their trial participation; thus meaning that only trial completers were invited. Though the high 12-month trial follow up rate of the intervention group means that very few individuals were excluded in this way (*n* = 6), it is possible that those who dropped out of the trial before this point had differing views. The decision to invite participants at the end of the trial was made to minimise the risk of the researchers who undertook trial assessments from being ‘unblinded’ during the intervention period. Future similar studies might benefit from putting in place mechanisms to enable non-completers to be invited, e.g. invitation by a non-blinded person who is not involved in trial assessments.

## Conclusions

This study provided useful data to inform the potential refinement of our intervention for further testing. We learned that individualised and collaborative healthy living interventions, which address barriers for people with psychosis (e.g.confidence, motivation) are acceptable. Acceptability may not solely relate to the achievement of weight loss, but may be derived from the wider psychosocial benefits that engagement in healthy living interventions can bring about. Whilst weight loss is an important outcome, these findings suggest that wider psychosocial outcomes are legitimate targets of intervention for lifestyle interventions in their own right. In particular, a refined intervention could target and measure change in the quality of participants’ social relationships, as well as psychological aspects including self-esteem, self-efficacy and mood. Evaluation of the acceptability of this intervention has highlighted the importance of these additional outcomes in enhancing acceptability. It has also identified potential mechanisms by which these outcomes might be targeted. These may include improving outcomes by providing opportunity for social interaction within the intervention program and by employing workers who are able to form high quality relationships with participants. Our findings indicate that non-clinical staff who have received appropriate lifestyle training are able to provide healthy living guidance and support to service users who are currently accessing early intervention services.

## Abbreviations

INTERACT Trial: INTERvention to encourage ACTivity improve diet and control weight gain
RCT: Randomised controlled trial
STR: Support Time Recovery worker
